# Random projection forest initialization for graph convolutional networks

**DOI:** 10.1016/j.mex.2023.102315

**Published:** 2023-08-05

**Authors:** Mashaan Alshammari, John Stavrakakis, Adel F. Ahmed, Masahiro Takatsuka

**Affiliations:** a Independent Researcher, Riyadh, Saudi Arabia; b School of Computer Science, The University of Sydney, NSW 2006, Australia; c Information and Computer Science Department, King Fahd University of Petroleum and Minerals, Dhahran, Saudi Arabia

**Keywords:** Deep learning, Graph convolutional network (GCN), Graph neural network (GNN), Random projection forests, Random Projection Forest Initialization for Graph Convolutional Networks

## Abstract

Graph convolutional networks (GCNs) were a great step towards extending deep learning to graphs. GCN uses the graph G and the feature matrix X as inputs. However, in most cases the graph G is missing and we are only provided with the feature matrix X. To solve this problem, classical graphs such as k-nearest neighbor (k-nn) are usually used to construct the graph G and initialize the GCN. Although it is computationally efficient to construct k-nn graphs, the constructed graph might not be very useful for learning. In a k-nn graph, points are restricted to have a fixed number of edges, and all edges in the graph have equal weights. Our contribution is Initializing GCN using a graph with varying weights on edges, which provides better performance compared to k-nn initialization. Our proposed method is based on random projection forest (rpForest). rpForest enables us to assign varying weights on edges indicating varying importance, which enhanced the learning. The number of trees is a hyperparameter in rpForest. We performed spectral analysis to help us setting this parameter in the right range. In the experiments, initializing the GCN using rpForest provides better results compared to k-nn initialization.

• Constructing the graph G using rpForest sets varying weights on edges, which represents the similarity between a pair of samples.Unlike k-nearest neighbor graph where all weights are equal.
• Using rpForest graph to initialize GCN provides better results compared to k-nn initialization. The varying weights in rpForest graph quantify the similarity between samples, which guided the GCN training to deliver better results.
• The rpForest graph involves the tuning of the hyperparameter (number of trees T). We provided an informative way to set this hyperparameter through spectral analysis.

## Graphical abstract

**Figure:**
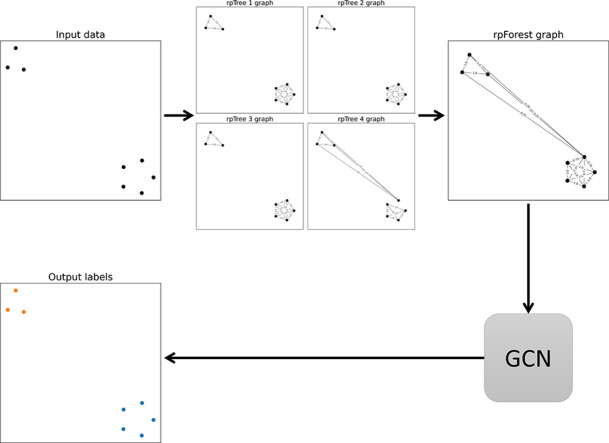

Specifications table

**Table:** 

Subject Area:	Computer Science
More specific subject area:	Graph Neural Network
Method name:	Random Projection Forest Initialization for Graph Convolutional Networks
Name and reference of original method:	N.A.
Resource availability :	https://github.com/mashaan14/RPTree-GCN

## Introduction

Convolutional neural networks (CNNs) proved to be effective in many applications. The convolution component in CNNs is only applicable to fixed grids like images and videos. Applying CNNs to non-grid data (graphs for example) can be useful for many applications. Graph Convolutional Networks (GCNs) [1] introduced a convolution component designed for graphs, where vertices are allowed to have a varying number of neighbors unlike fixed grids. GCNs were used in several application such as sentiment analysis [2], computer vision tasks [3] and ranking gas adsorption properties [4].

Because the edges arrangements are different from one graph to another, GCN needs the adjacency matrix A to perform the convolutions. The GCN performs two changes to the input adjacency matrix: 1) it adds self-loops to include the vertex own feature vector into the convolution, and 2) it normalizes the adjacency matrix using the degree matrix D to avoid favoring vertices with many edges. These two changes were embedded in the convolution function of GCN. Researchers have improved the GCN algorithm in different ways such as: increasing the depth of GCN [5], implementing attention mechanism [4], [6], or replacing self-loops with trainable skip connection [7]. Our focus is on creating an adjacency matrix in an unsupervised way.

GCN can classify graph vertices efficiently if the adjacency matrix is given, but it cannot create an adjacency matrix from scratch. This opens a new research track on how to create an adjacency matrix for GCN. An obvious solution is to use traditional methods to create the adjacency matrix such as: fully connected graph, k-nearest neighbor graph, and ϵ-neighborhood graph [8]. The fully connected graph grows exponentially with the number of vertices (n), which makes k-nn and ϵ-graphs more appealing. But if we compare k-nn and ϵ-graphs, we can see that k-nn graphs can be implemented using efficient data structures such as kd-trees.

Franceschi et al. proposed a bi-level optimization for GCN graphs [9]. They used a number of sample graphs to train the GCN and based on the validation error they modified the original adjacency matrix. Although the method proposed by Franceschi et al. is effective in learning the adjacency matrix, it still uses the k-nearest neighbor graph. k-nn graphs have two problems. First, all vertices in a k-nn graph are restricted to k edges, this would limit the ability of vertices to connect to more similar neighbors. Second, all edges in a k-nn graph are assigned equal weights, which gives them the same level of importance when passed through a GCN.

We present a new method to construct the adjacency matrix for GCN. We used random projection forests (rpForest) [10], [11] to construct the adjacency matrix. An rpForest is a collection of random projection trees (rpTree). rpTrees use random directions to partition the data points into tree nodes [12], [13], [14]. A leaf node in an rpTree represents a small region that contains similar points. We connect all points in a leaf node in each rpTree. If an edge keeps persisting over multiple rpTrees it will be assigned a higher weight. This would solve the equal weights problem in k-nn graphs. Also, since leaf nodes can have a varying number of points, this would allow points to connect to more or less neighbors depending on the density inside the leaf node. The experiments showed that a GCN with rpForest initialization performed better than a GCN with k-nn initialization. Our contributions are:

• Initializing GCN using a graph based on rpForest, that allows points to connect to a varying number of neighbors and assigns weights proportionate to the edge’s occurrence in the rpForest.
• Providing a spectral analysis to set the hyperparameter (number of trees T) in rpForest.

## Related work

This section discusses the recent advancements in graph convolutional networks, graph construction methods, and random projection forests. These three topics form the basis of our proposed method.

### Graph convolutional networks (GCNs)

The successful application of convolutional neural network (CNN) on imagery data has stimulated research to extend the convolution concept beyond images. Images can be viewed as a special case of graphs where edges and vertices are ordered on a fixed grid. The problem with applying convolutions on graphs is that vertices have a varying number of edges. GCN extended the convolution to graphs by performing three steps: 1) feature vectors are averaged within the node’s local neighborhood, 2) the averaged features are transformed linearly, and 3) a nonlinear activation is applied to the averaged features [15].

Wu et al. [16] have categorized graph convolutional network methods into two categories: 1) spectral-based GCNs and 2) spatial-based GCNs. Spectral-based GCNs rely on spectral graph theory. The intuition is that the graph Laplacian carries rich information about graph geometry. A symmetric graph Laplacian is defined as $L=D^{-\frac{1}{2}}LD^{-\frac{1}{2}}=I-D^{-\frac{1}{2}}AD^{-\frac{1}{2}}$ [8], [17]. D is the degree matrix in which the diagonal shows the degree of each vertex $D_{ii}=\sum_{j}A_{ji}$. The graph Fourier transform projects an input graph signal x to an embedding space, where the basis are formed by eigenvectors of the normalized graph Laplacian L. The process of graph convolution can be thought of as convoluting an input signal x with a filter $g_{\theta }=diag\left(\theta \right)$ as shown in Eq. (1):

(1) $$g_{\theta }*x=Ug_{\theta }U^{⊤}x,$$

where $\theta \in R^{n}$ is a parameter that controls the filter g and U is the matrix of eigenvectors ordered by eigenvalues. Bruna et al. represented the filter g as a set of learnable parameters [18]. Henaff et al. [19] extended the model proposed by Bruna et al. [18] to datasets where graphs are unavailable. Wu et al. [16] have identified three limitations for spectral-based GCNs: 1) any change in the graph structure would change the embedding space, 2) the learned filters are domain specific and cannot be applied to different graphs, 3) they require an eigen decomposition step, which is computationally expensive $O(n^{3})$.

Although the graph spectrum provides rich information, avoiding the eigen decomposition step will be a huge boost in terms of performance. Hammond et al. [20] proposed an approximation of the filter $g_{\theta }$ via Chebyshev polynomials $T_{k}\left(x\right)$, which was deployed into GCN by Defferrard et al. [21]. The Chebyshev polynomials are recursively defined as:

(2) $$T_{k}\left(x\right)=2xT_{k-1}\left(x\right)-T_{k-2}\left(x\right),$$

with $T_{0}\left(x\right)=1$ and $T_{1}\left(x\right)=x$. This formulation would allow us to perform the convolution on graphs as:

(3) $$g_{\theta^{\prime }}*x=\sum_{k=0}^{K}\theta_{k}^{\prime }T_{k}\left(\tilde{L}\right)x,$$

with $\tilde{L}=\frac{2}{\lambda_{\max}}L-I_{N}$. This K localization is a Kth-order neighborhood, which means that it depends on the nodes that are K steps away from the central node. This graph convolution was simplified by Kipf and Welling [1]. They limited the layer-wise convolution operation to K=1, and set $\lambda_{\max}=2$. Using these settings, the graph convolution in Eq. (3) can be simplified to:

(4) $$g_{\theta^{\prime }}*x=\theta_{0}x-\theta_{1}D^{-\frac{1}{2}}AD^{-\frac{1}{2}}x.$$

The time complexity for GCN is $O(LAd+LNd^{2})$, where L is the number of layers, A is the adjacency matrix, d is the number of features, and N is the number of nodes [22].

### Graph construction

All methods explained in the previous section assume the graph G(V,E) to be already constructed. But this is not the case in many practical applications, where only the feature matrix X is provided. When a graph is missing, the most used way to construct it is to use the Gaussian heat kernel [23]:

(5) $$A_{ij}=\exp\left(\frac{-d^{2}\left(i,j\right)}{2\sigma^{2}}\right),$$

where $d^{2}\left(i,j\right)$ is the distance between the samples i and j. The problem with the heat kernel is that it heavily depends on the selection of the scaling parameter $\sigma^{2}$, and usually the user has to try different values and selects the best one. This was improved by the self-tuning diffusion kernel [24]:

(6) $$A_{ij}=\exp\left(\frac{-d^{2}\left(i,j\right)}{\sigma_{i}\;\sigma_{j}}\right),$$

where $\sigma_{i}$ is the distance from the point $x_{i}$ to its Kth neighbor. Zelnik-manor and Perona have set K=7 [24].

Constructing a graph using these two approaches requires performing pairwise comparisons, which means computations in order of $O(n^{2})$. To avoid these computations, one could use a more efficient data structure. k-nearest neighbor graphs are usually implemented using k-dimensional trees (also known as kd-trees) [25]. kd-trees start by selecting the dimension with maximum dispersion. Along that dimension they split at the median and place whatever less than the median in the left child and whatever greater than the median in the right child. After several recursive executions, the kd-tree algorithm scans the leaf nodes and returns the k-nearest neighbors. The k-nearest neighbor graph is defined as:

(7) $$A_{ij}=\left\{ \begin{array}{cc} 1, & j\in knn(i),\\ 0, & \text{otherwise} \end{array}\right..$$

We can identify two problems with a k-nearest neighbor graph: 1) it assigns equal weights on edges which gives all edges the same importance, and 2) it restricts all points to have k edges regardless of their position in the feature space.

### Random projection forests (rpForests)

**Fig. 1:**
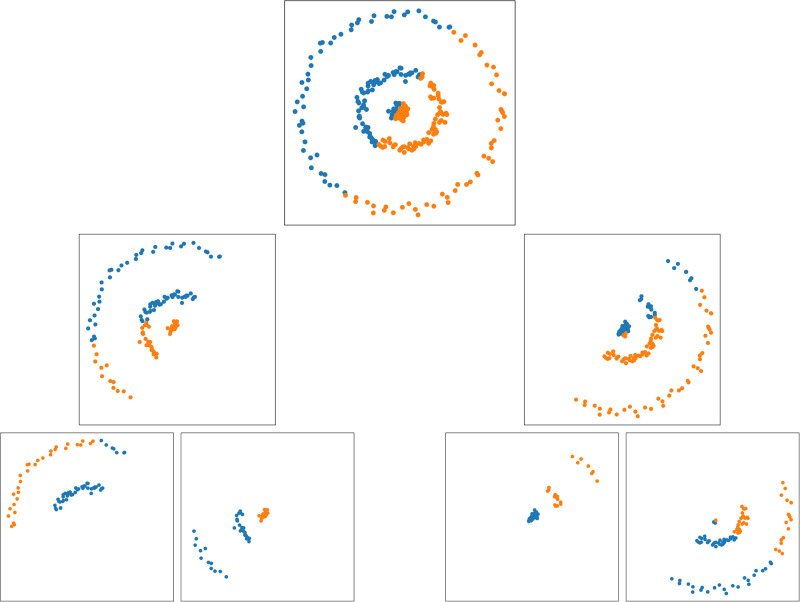
An example of running rpTree algorithm on points in 2D. At each node in the tree, a random direction is selected, and all points are projected onto it. Points are split at the median, where points less than the median (points in blue) are placed in the left child, and points larger than the median (points in orange) are placed in the right child. (Best viewed in color). (For interpretation of the references to colour in this figure legend, the reader is referred to the web version of this article.)

Random projection forest (rpForest) is a collection of random projection trees (rpTrees) [12], [26]. rpTrees use the same principle as kd-trees, that is partitioning the feature space and placing points in a binary tree. The difference is that kd-tree splits along the existing dimensions, while rpTree splits along random directions. In rpTrees, the root node contains all the data points, and the leaf nodes contain disjoint subsets of these data points. Each internal node in rpTree holds a random projection direction $\vec{r}$ and a scalar split point c along that random direction. Fig. 1 shows an example of rpTree.

The most common use of rpTrees is performing k-nearest neighbor search. Yan et al. [11] used rpForest (i.e., a collection of rpTrees) to perform k-nn search. They modified the splitting rule by selecting three random directions, then project onto the one that yields the maximum dispersion of points. rpTree was used in anomaly detection application by Chen et al. [27]. They also modified the splitting rule by examining if the points form two Gaussian components, they will split these two components into left and right nodes. Tavallali et al. [28] proposed a k-means tree, which outputs the centroids of clusters. All these modifications on rpTree are supported by the empirical evidence provided by Ram and Gray [29]. They stated that the best performing binary space-partitioning trees are the ones that have better vector quantization and large partition margins. But these modifications add extra computations to the rpTree algorithm.

## Proposed method

Our proposed method has two components: a neural network and a graph construction component. The next section explains the neural network component, followed by two sections discussing the graph construction component.

### GCN and LDS

Graph convolutional networks (GCNs) are used for semi-supervised node classification. A GCN propagation rule at the first layer is defined as:

(8) $$f\left(X,A\right)=ReLU\left(AXW^{\left(0\right)}\right),$$

where A is the adjacency matrix, ReLU is the activation function f(x)=max(0,x), and $W^{\left(0\right)}$ is the weight matrix for the first neural network layer. Two problems arise from this definition. First, the node’s own feature vector is not included since A has zeros on the diagonal. This can be solved by allowing self-loops, and rewrite the adjacency matrix to be $\tilde{A}=A+I$. The second problem is the normalization of the adjacency matrix, which can be solved by normalizing using the degree matrix D. The adjacency matrix becomes $\hat{A}=D^{-\frac{1}{2}}\tilde{A}D^{-\frac{1}{2}}$. By applying these changes, the GCN propagation rule in Eq. (8) can be rewritten as:

(9) $$f\left(X,A\right)=ReLU\left(\hat{A}XW^{\left(0\right)}\right).$$

The most common architecture is a two-layer GCN, which can be defined using the following formula:

(10) $$f\left(X,A\right)=softmax\left(\hat{A}\;ReLU\left(\hat{A}XW^{\left(0\right)}\right)W^{\left(1\right)}\right).$$

Franceschi et al. [9] proposed LDS method to learn the adjacency matrix A. It stands for Learning Discrete Structures (LDS). They used k-nearest neighbor graph to initialize the Graph Convolutional Networks (GCNs). The methodology involves four steps: initialization, sampling, inner optimization, and outer optimization. First, a parameter θ is initialized to be the adjacency matrix of k-nn graph and run GCN once to initialize its parameters. Then, the method iteratively sample graphs from θ to optimize for GCN parameters (inner optimizer) and θ (outer optimizer) in a bilevel optimization.

In our experiments, we used both methods GCN and LDS to evaluate their performance when we initialize them using different graphs. The initializations we used in the experiments are k-nearest neighbor graph initialization and random projection forest (rpForest) initialization.

### k-nearest neighbor initialization

GCN needs a graph to perform the convolutions. A common choice is to use the k-nearest neighbor graphs to initialize the GCN. In a k-nn graph, each point is connected to its nearest k neighbors. Intuitively, the adjacency matrix contains k×n nonzero entries since we have n points and each one of them has k edges. A formal definition for k-nn graphs is given in Eq. (7).

**Fig. 2:**
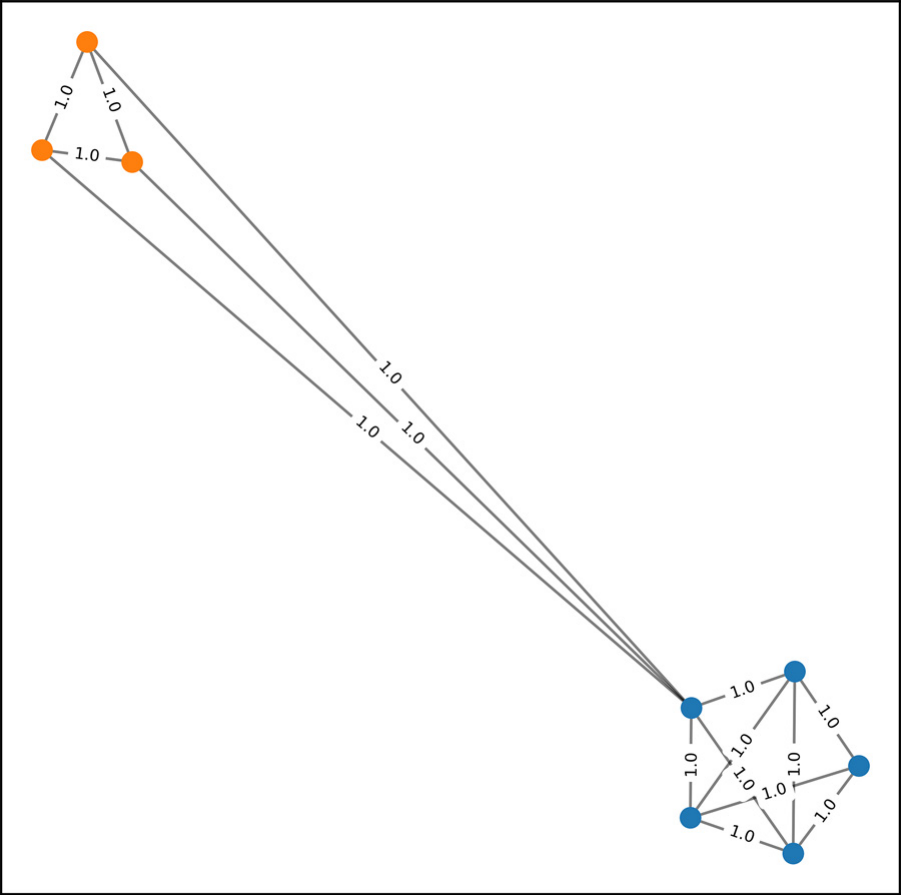
A k-nn graph with k=3; all edges were assigned equal weights even the ones connecting two different classes. (Best viewed in color). (For interpretation of the references to colour in this figure legend, the reader is referred to the web version of this article.)

An example of k-nearest neighbor graph is shown in Fig. 2. In that figure, we have three points in the blue class and five points in the orange class. The graph was constructed with k=3. Note that some edges connect two points from two different classes. The existence of these edges is undesirable because they could confuse the classifier. But in an unsupervised graph construction, these edges are sometimes unavoidable. The solution is to assign a small weight on these edges connecting two different classes. Unfortunately, we cannot do that in k-nn graphs because all edges get an equal weight.

k-nn provides a fast initialization for the graph convolution networks (GCN). But it assigns equal weights to all edges which gives the edges spanning two classes the same importance as edges connecting one class. To avoid this problem, we need a graph construction scheme that assigns small weights on edges connecting two classes.

### rpForest initialization

The random projection tree (rpTree) is a binary space-partitioning tree. The root node contains all points in the dataset. For each node in the tree, the method picks a random direction $\vec{r}$. The dimensions of $\vec{r}$ is $R^{d-1}$, where d is the number of dimensions in the dataset. All points in the tree node get projected onto $\vec{r}$. Then, a split point c is selected randomly between $\left[\frac{1}{4},\frac{3}{4}\right]$ along $\vec{r}$. In the projection space, if a point is less than c it is placed in the left child, otherwise it is placed in the right child. rpTrees are particularly useful for k-nearest neighbor search. But in this paper, we are going to use them for graph construction.

A collection of rpTrees is called rpForest. rpForests were proposed by Yan et al. [10], [11] and they applied it in spectral clustering similarity and k-nearest neighbor search. We used rpForests to construct a graph and use it as an input to the GCN. rpForests helped us to overcome two problems we identified with k-nn graphs. The problem of equal weights on edges, and the problem of restricting points to a fixed number of neighbors. We constructed a number of rpTrees. Then, we connect all points falling into the same leaf node. The intuition is simple, if a pair of points fall into the same leaf node in all rpTrees, they will be connected with the maximum weight. Otherwise, the weight on the edge connecting them will be proportionate to the number of the leaf nodes they fall in together. Also, the points will not be restricted to a fixed number of edges because the number of points varies from one leaf node to another.

**Fig. 3:**
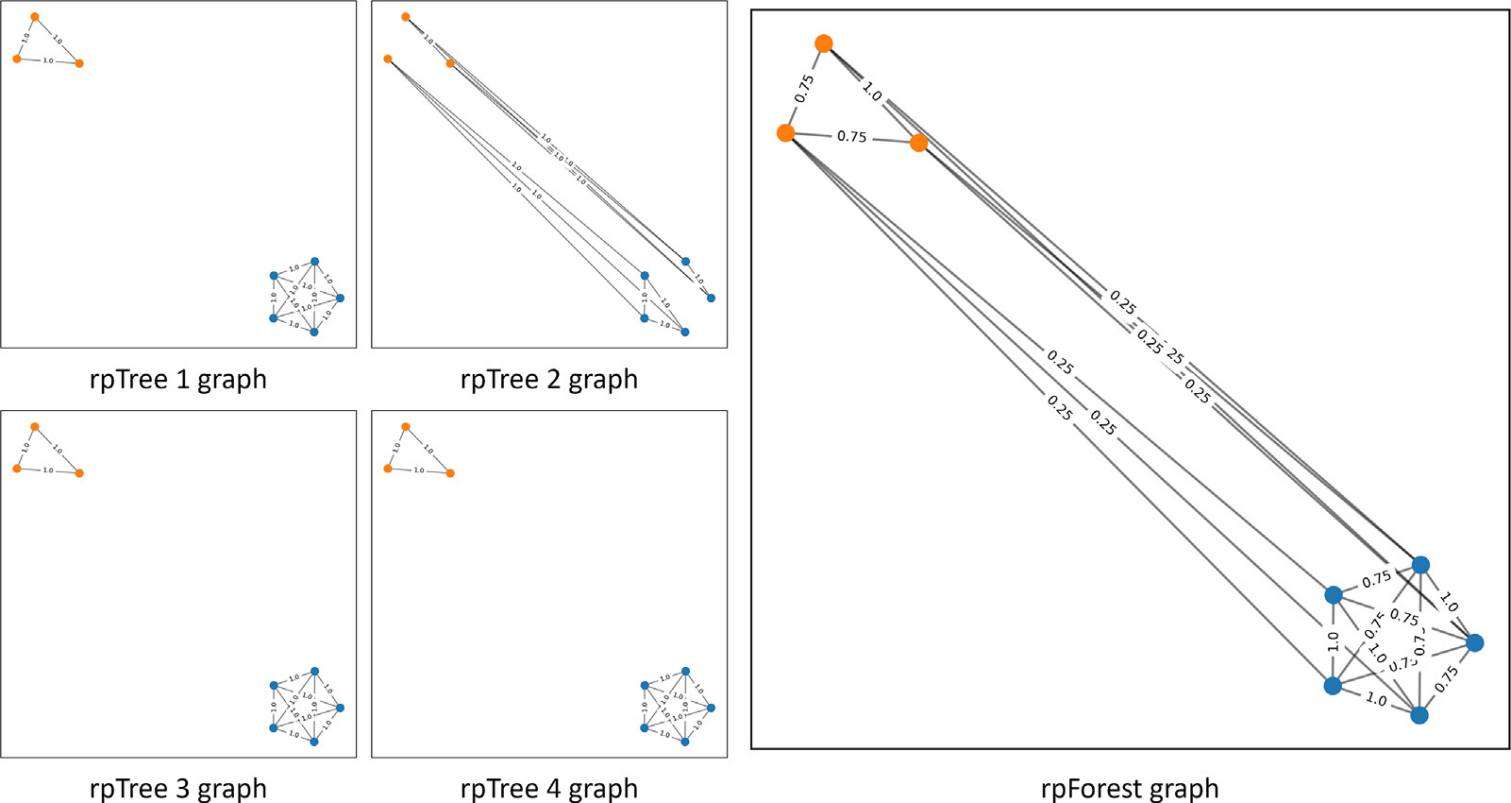
An rpForest graph with T=4; edges connecting the two classes were assigned a small weight (0.25) because they only appear in one tree out of four. (Best viewed in color). (For interpretation of the references to colour in this figure legend, the reader is referred to the web version of this article.)

Fig. 3 illustrates how did we construct a graph using rpForest. In that figure we used four rpTrees each of which has two levels meaning we only perform the split once. Apart from (rpTree 2), all trees have split the two classes into two different leaf nodes. The final graph aggregates all edges in the trees. The edges connecting the two classes were assigned a small weight (0.25) because they only appear in one tree out of four. Edges connecting points from the same class were assigned higher weights (1 or 0.75) because they either appear in all trees or in three out of four trees.

## Experiments and discussions

We designed our experiments to test how k-nn and rpForest graphs affect the performance of GCN [1] and LDS [9]. Unlike GCN, LDS iteratively improves the original graph based on the validation error. For the k-nn graph, we used the same settings used by the LDS algorithm, where k was set to be 10. For the rpForest graph, we set the number of trees T to be 10. In the next section we provide an empirical examination showing why it is safe to use T=10 as the number of trees. The number of layers in GCN was kept less than 4 layers, to prevent a drop in the performance [5]. We used two evaluation metrics: 1) the test accuracy was used to evaluate the performance, and 2) the number of edges in the graph was used to evaluate the storage efficiency.

We modified the original python files provided by GCN and LDS, to include the code for rpForest graph. We used a different font for the names of datasets. The name of a dataset is written as dataset. The code used to produce the experiments is available on https://github.com/mashaan14/RPTree-GCN. All experiments were coded in python 3 and run on a machine with 20 GB of memory and a 3.10 GHz Intel Core i5-10500 CPU.

### Using spectral analysis to set the number of trees

We are constructing the graph out of the leaf nodes in the rpForest. The number of rpTrees T is a hyperparameter in rpForest. Tuning T highly influences the outcome of rpForest. We can identify two problems that could occur from different values of T. The first problem occurs when T is set to a low value, which risks feeding a disconnected graph to the GCN. A disconnected graph could mean one of the classes is not connected, which negatively affects the performance of the GCN. The second problem occurs when T is set to a high value. This will lead to a graph with so many edges, which could affect the memory efficiency of our method.

**Fig. 4:**
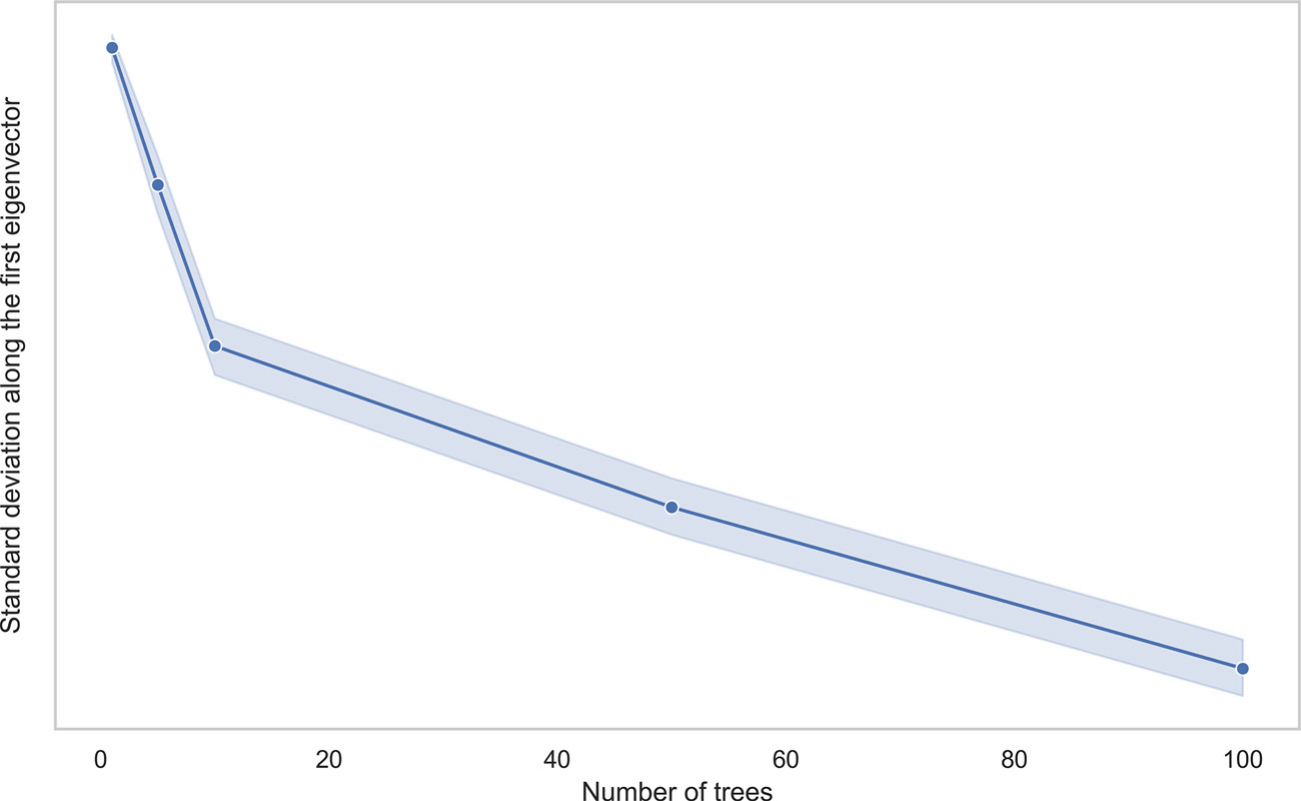
Measuring the standard deviation of points along the smallest eigenvector $v_{0}$; T=10 represents an elbow point. (For interpretation of the references to colour in this figure legend, the reader is referred to the web version of this article.)

**Fig. 5:**
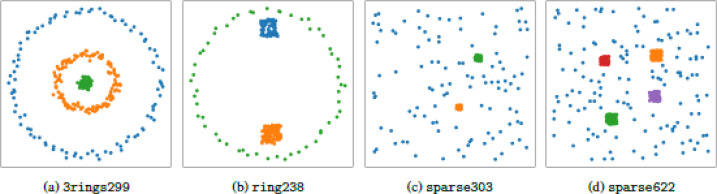
2-dimensional datasets used in the experiments. (For interpretation of the references to colour in this figure legend, the reader is referred to the web version of this article.)

Spectral analysis provides an elegant way to check the graph connectivity. The eigenvector $v_{0}$ associated with the smallest eigenvalue $\lambda_{0}$ of the graph Laplacian L, that eigenvector should be constant. This was stated in (Proposition 2) by von Luxburg [8]. She wrote “*In a graph consisting of only one connected component we thus only have the constant one vector* 1 *as eigenvector with eigenvalue 0*”. So, we used the standard deviation of points along the smallest eigenvector $v_{0}$ to see if the graph is connected or not. If the graph is connected (i.e. it contains a one connected component) the standard deviation will be small. We used the ring238 dataset, which is a 2D dataset shown in Fig. 5. In Fig. 4, there is a clear elbow point at T=10, which means the graph becomes connected from this point onwards.

The second problem related to the number of trees T is setting T to a large value, which affects the memory efficiency. Naturally if we start with a low T, some edges will be missing. These edges will be created as we increase T. At some point, the graph will have the same edges even if we increase T. Based on our empirical analysis we set T=10 in all experiments.

### GCN and LDS using k-nn and rpForest graphs

In this experiment, we compared the performance of GCN and LDS using k-nn and rpForest graphs. We used four 2-dimensional datasets. These 2D datasets are shown in Fig. 5 with their class labels. We also used four datasets retrieved from scikit-learn library [30]. For train and test splits we used the same settings in LDS paper [9]. These settings are shown in Table 1.

**Table 1:** Summary statistics of the datasets.

Name	Samples	Features	|y|	Train/Valid/Test
3rings299	299	2	3	10 / 20/ 279
rings238	238	2	3	10 / 20 / 218
spares303	303	2	3	10 / 20 / 283
spares622	622	2	5	10 / 20 / 602
Iris	150	4	3	10 / 20 / 130
Wine	569	30	2	10 / 20 / 158
Cancer	569	30	2	10 / 20 / 539
Digits	1797	64	10	50 /100 / 1,647

**Fig. 6:**
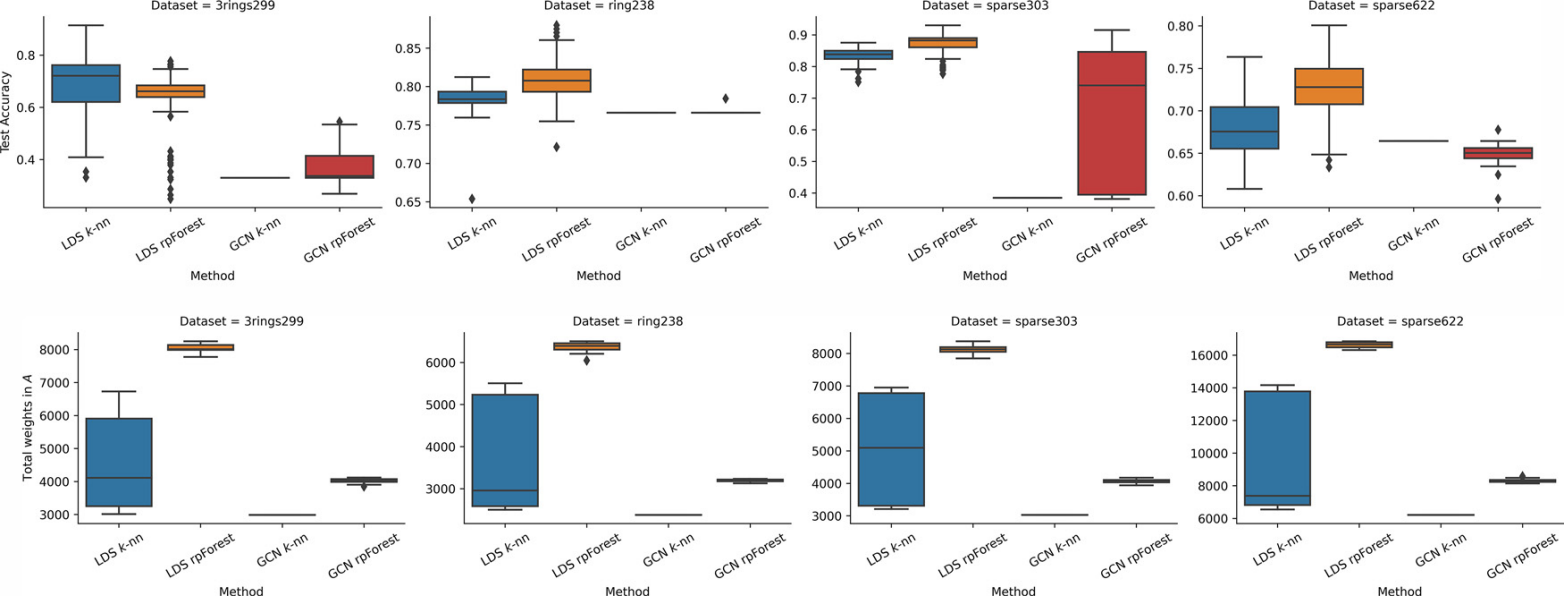
Running LDS and GCN using k-nn and rpForest graph on 2D datasets; (top) test accuracy; (bottom) total weights in the adjacency matrix A. (For interpretation of the references to colour in this figure legend, the reader is referred to the web version of this article.)

Fig. 6 shows the results of running GCN and LDS on 2-dimensional datasets. In general, we can see LDS performed better than GCN, whether it is using k-nn graph or rpForest graph. This can be explained by how these two methods work. GCN takes the graph and runs it through the deep network, it cannot modify the graph by adding or removing edges. On the other hand, LDS uses the validation error to modify the graph by keeping the edges that minimize the validation error. Of course, LDS needs more time than GCN.

LDS performed better when given a graph based on rpForest compared to k-nn graph. But this was not the case with 3rings299 dataset, when the linear line split by rpForest breaks the two rings in 3rings299 dataset. Also, we observed that rpForest graph has improved the performance of GCN. The total weights in the adjacency matrix A gives us a hint about memory efficiency. Graphs in LDS have more weight than GCN, because LDS keeps modifying the graph by adding more edges. Another thing to highlight is rpForest graphs have more edges than k-nn graphs.

**Fig. 7:**
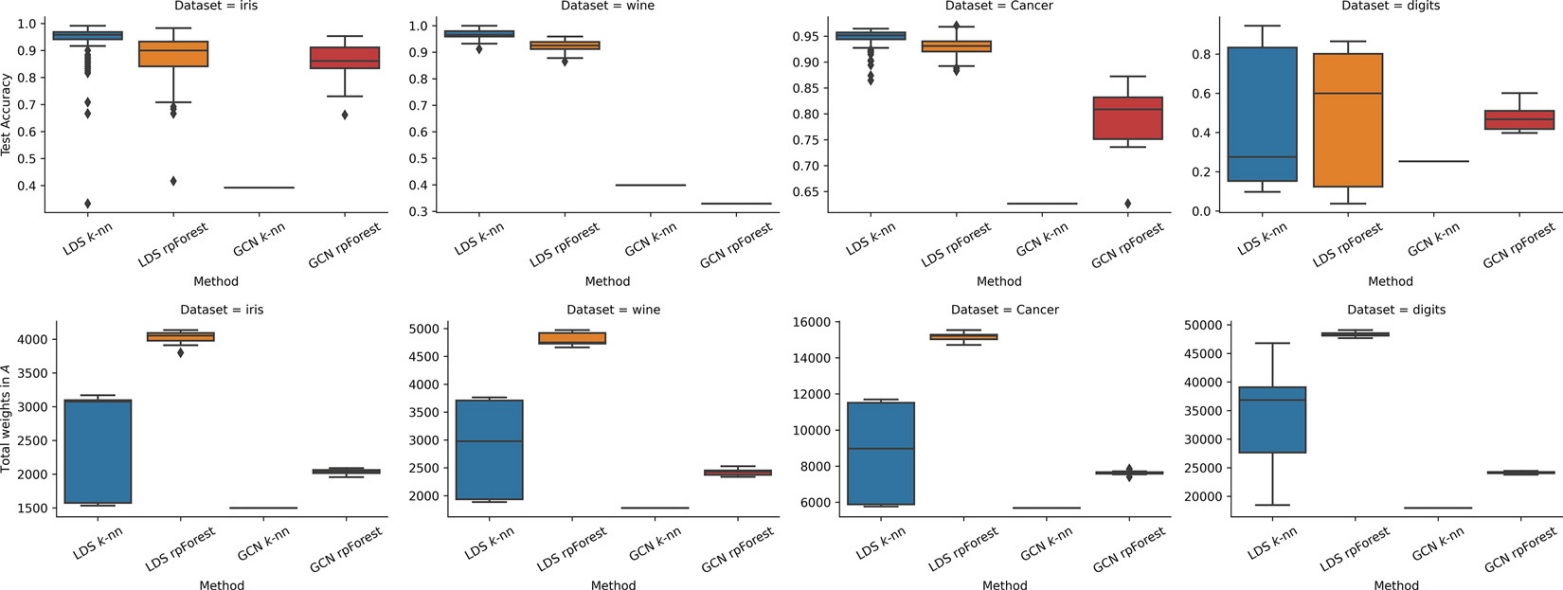
Running LDS and GCN using k-nn and rpForest graph on scikit-learn datasets; (top) test accuracy; (bottom) total weights in the adjacency matrix A. (For interpretation of the references to colour in this figure legend, the reader is referred to the web version of this article.)

The results of experiments on scikit-learn datasets are shown in Fig. 7. GCN test accuracy was very close to the one delivered by LDS in iris and digits, even though LDS has the ability to modify the graph. Another observation is that when a GCN is fed an rpForest graph it performs better compared to k-nn graph. For the total weights metric, we had the same observation across all datasets. LDS requires more storage especially when we feed it an rpForest graph, whereas GCN requires less storage.

### LDS using rpForest graph with extra edges

**Fig. 8:**
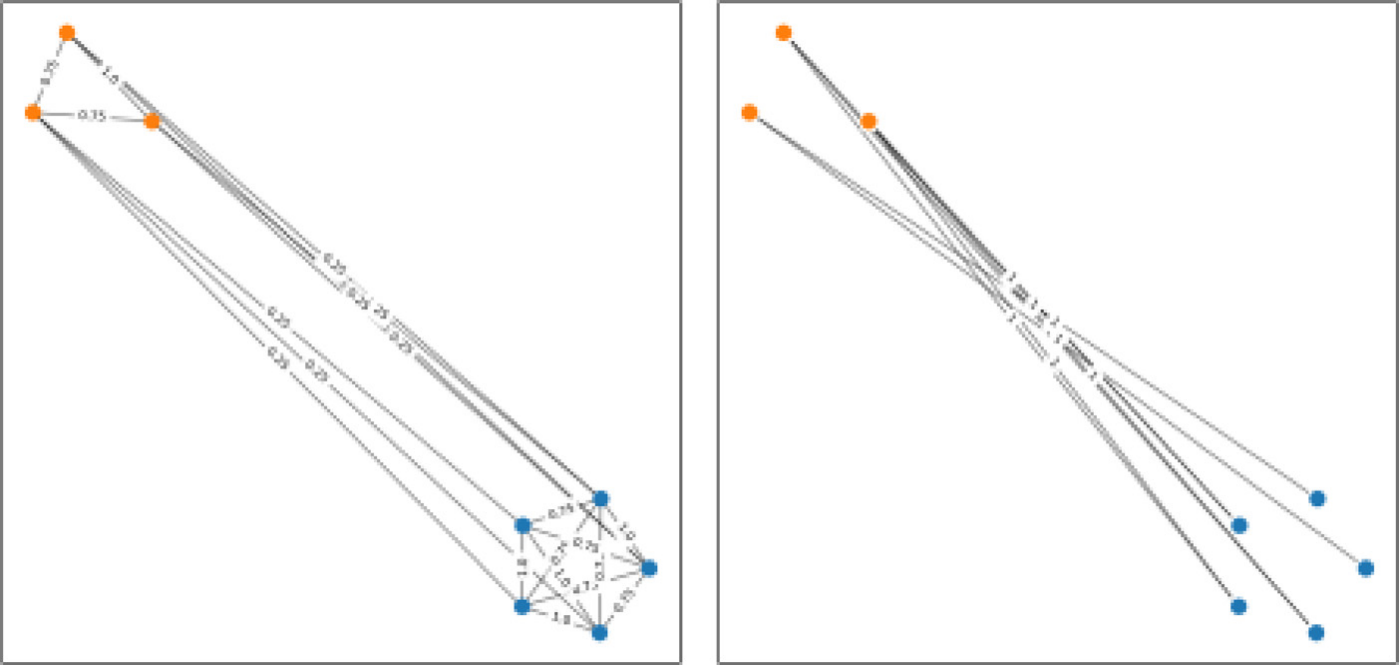
(left) an rpForest graph; (right) edges that did not appear in the rpForest graph. (For interpretation of the references to colour in this figure legend, the reader is referred to the web version of this article.)

The rpForest graph connects only the points from the same leaf node. In this experiment, we investigate if we add some edges between points from different leaf nodes, would this improve the performance. Fig. 8 shows an example of rpForest graph and the edges that did not appear in the rpForest graph. We want to examine if we take a percentage of these edges that did not appear in the rpForest graph, would that increase the connectivity and consequently improve the performance.

**Fig. 9:**
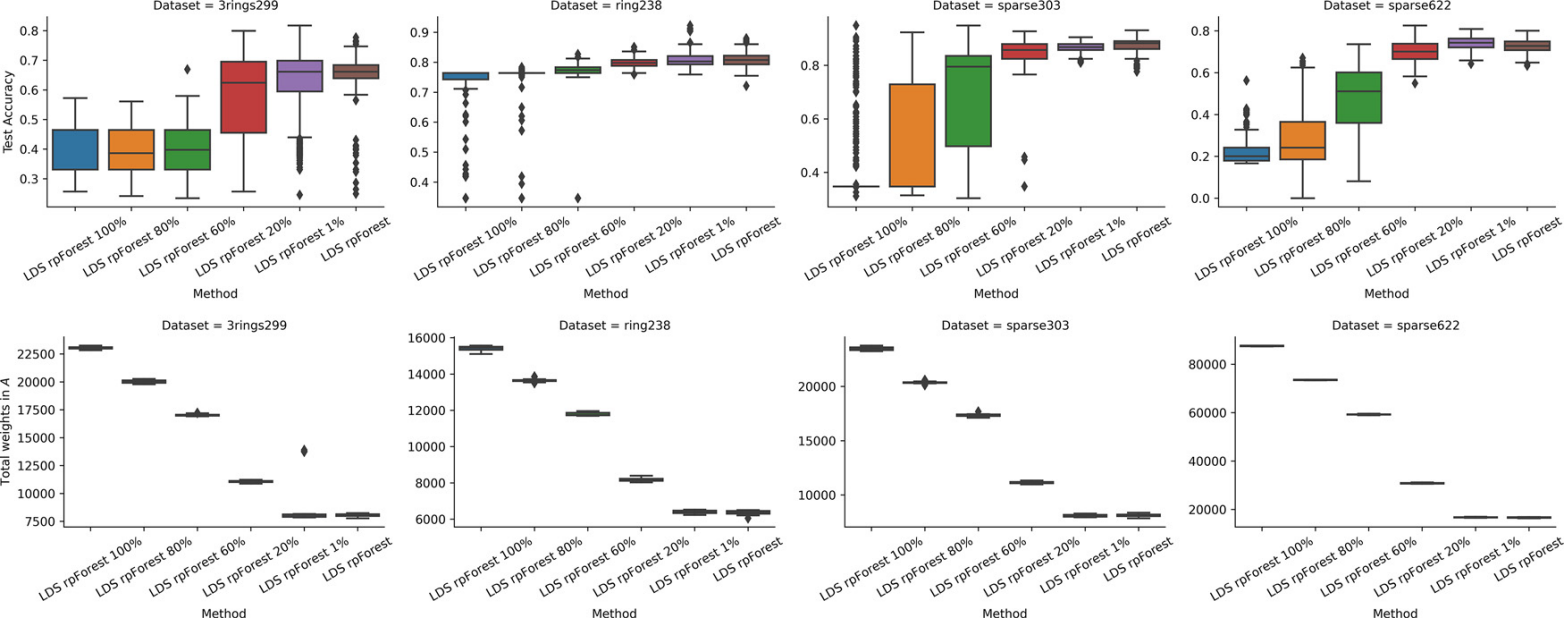
Running LDS using rpForest with extra edges; the percentage on the x-axis represents the percentage of extra edges; (top) test accuracy; (bottom) total weights in the adjacency matrix A. (For interpretation of the references to colour in this figure legend, the reader is referred to the web version of this article.)

By looking at Fig. 9, which shows LDS test accuracies using extra edges, we can see that these extra edges did not improve the performance. Even at the most extreme case when we included 100% of these edges, the performance dropped by 50% in some datasets. The memory footprint of these extra edges was very large. These findings emphasize on the ability of rpForest to find the most important edges for classification.

One advantage for our method is assigning weights proportionate to the edge’s occurrence in the rpForest. This allows non-equal weights across the graph. A potential application for our method is the analysis of complex networks in the brain [31]. This is a research area in neuroscience that studies the complex connectivity on neuronal circuit dynamics. The functional connectivity between brain areas can be modeled as edges on a graph. These edges must have some varying weights, which is provided by our method.

## Conclusion

Graphs are useful in modeling real-world relationships. That is why researchers were keen on extending deep learning to graphs. One of the successful applications of deep learning on graphs is graph convolutional networks (GCNs). The problem with GCN is that it needs the graph prepared beforehand. In most cases, the graph must be constructed from the dataset. A common choice to construct the graph is to use k-nearest neighbor graph. But k-nn assigns equal weights on all edges, which gives all edges the same importance during deep learning training.

We present a graph based on random projection forests (rpForest) with varying weights on edges. The weight on the edge was set proportional to how many trees it appears on. The number of trees is a hyperparameter in rpForest that needs careful tuning. We performed spectral analysis that helps us to set this parameter within the right range. The experiments revealed that initializing GCN using rpForest delivers better accuracy than k-nn initialization. We also showed that the edges provided by rpForest are the best for learning and adding extra edges did not improve the performance.

For future work, we can try a different weight assignment strategy other than average, a Euclidean distance for example. Another potential extension to our work could be investigating how different binary space-partitioning trees would affect the performance of the GCN. Also, it is important to examine how rpForest graph would perform in different methods of graph neural networks (GNNs).

## CRediT authorship contribution statement

**Mashaan Alshammari:** Conceptualization, Formal analysis, Investigation, Methodology, Project administration, Software, Visualization, Writing – original draft. **John Stavrakakis:** Conceptualization, Formal analysis, Investigation, Methodology, Validation, Writing – review & editing. **Adel F. Ahmed:** Conceptualization, Formal analysis, Supervision, Validation, Writing – review & editing, Funding acquisition. **Masahiro Takatsuka:** Conceptualization, Formal analysis, Supervision, Validation, Writing – review & editing.

## Declaration of Competing Interest

The authors declare that they have no known competing financial interests or personal relationships that could have appeared to influence the work reported in this paper.

## Data Availability

I have shared a link to my code/data.
